# Black-blood T_1_ mapping at 3T: Reduced partial-voluming using adiabatic MSDE preparation

**DOI:** 10.1186/1532-429X-18-S1-W5

**Published:** 2016-01-27

**Authors:** Sebastian Weingärtner, Nadja M Messner, Frank G Zoellner, Lothar R Schad

**Affiliations:** University Medical Center Mannheim, Heidelberg University, Computer Assisted Clinical Medicine, Mannheim, Germany

## Background

Myocardial T_1_ mapping in pathologies with decreased myocardial wall thickness such as dilated cardiomyopathy (DCM) is strongly impaired by partial-voluming from the neighboring blood pools [Kellman et al., JCMR2014].

Significant differences between the T_1_ times in myocardium and blood lead to decreased accuracy in the presence of partial-voluming. This causes sensitivity to the region-of-interest (ROI), compromising the inter-observer reproducibility.

The aim of this work is to study the use of blood-signal suppression using a motion-sensitized driven equilibrium (MSDE) [Wang et al., MRM2007] magnetization preparation in order to reduce partial-voluming in myocardial T_1_ mapping.

## Methods

**Figure 1:**
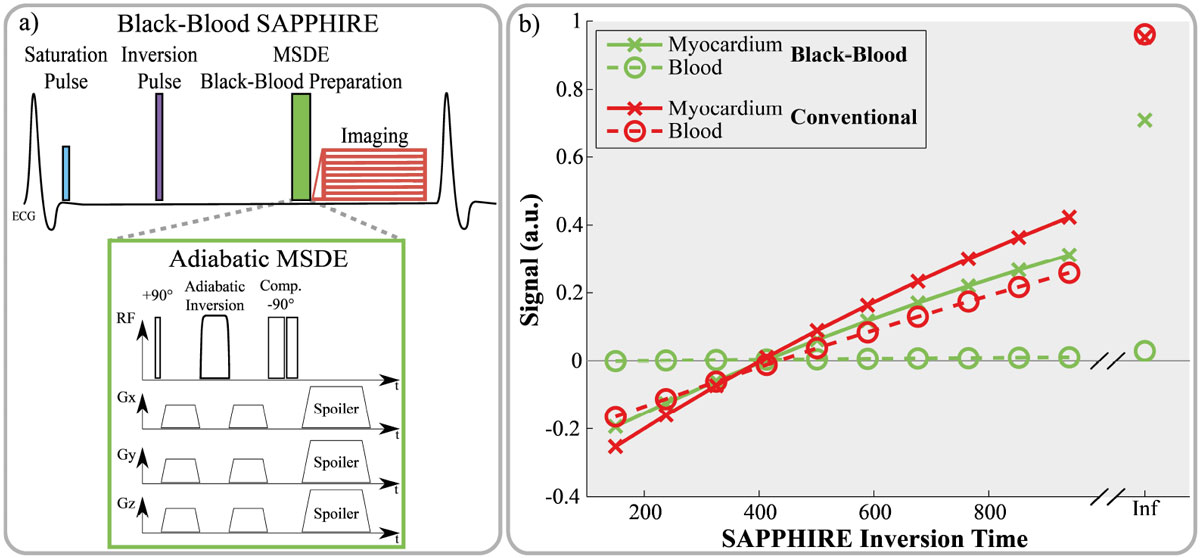
**a) Sequence diagram of the SAPPHIRE black-blood T**_**1**_ **mapping sequence**. An adiabatic MSDE preparation is inserted directly before the imaging pulses. In MSDE, blood-signal suppression is caused by symmetric dephasing gradients before and after a refocusing pulse, causing incomplete refocusing of moving tissue and greatly increasing the blood/myocardium contrast. b) Simulated magnetization signal at the various inversion times of a SAPPHIRE sequence. High signal is observed in conventional T_**1**_mapping from the blood pools despite the long T_**1**_time. Black-blood SAPPHIRE shows almost complete suppression of the blood signal for the trade-off against a slightly decreased dynamic range in the myocardial signal.

An adiabatic MSDE preparation module was added directly before the imaging pulses of a SAPPHIRE sequence [Weingärtner et al., MRM2014] (Fig. 1). The preparation consists of a rectangular tip-down pulse, an adiabatic BIREF1 refocusing pulse, a composite tip-up pulse and motion-sensitizing gradients before and after refocusing. The MSDE parameters were TE_MSDE_ = 11 ms, gradients: amplitude = 16 mT/m, duration = 2 ms.

6 healthy volunteers (25 ± 6 y; 4 M) were scanned using conventional and black-blood T_1_ mapping on a 3T MR Scanner (Siemens Skyra). T_1_ mapping was performed using a bSSFP imaging readout with the following parameters: TE/TR/α = 1.0 ms/2.9 ms/35°, FOV/res = 440 × 375 mm²@1.7 × 1.7 mm², sl.th. = 8 mm, GRAPPA = 2, Partial-Fourier = 6/8, bw = 1085 Hz/px.

A three parameter model was used for T_1_ fitting, avoiding potential quantification inaccuracies caused by the recovery curve modulation through the MSDE preparation. T_1_ times, the average thickness and the apparent in-plane area of the myocardium were quantified in the T_1_ maps using manually drawn ROIs. Furthermore, cross myocardial T_1_ times were analyzed from the endo- to the epicardial border.

## Results

**Figure 2:**
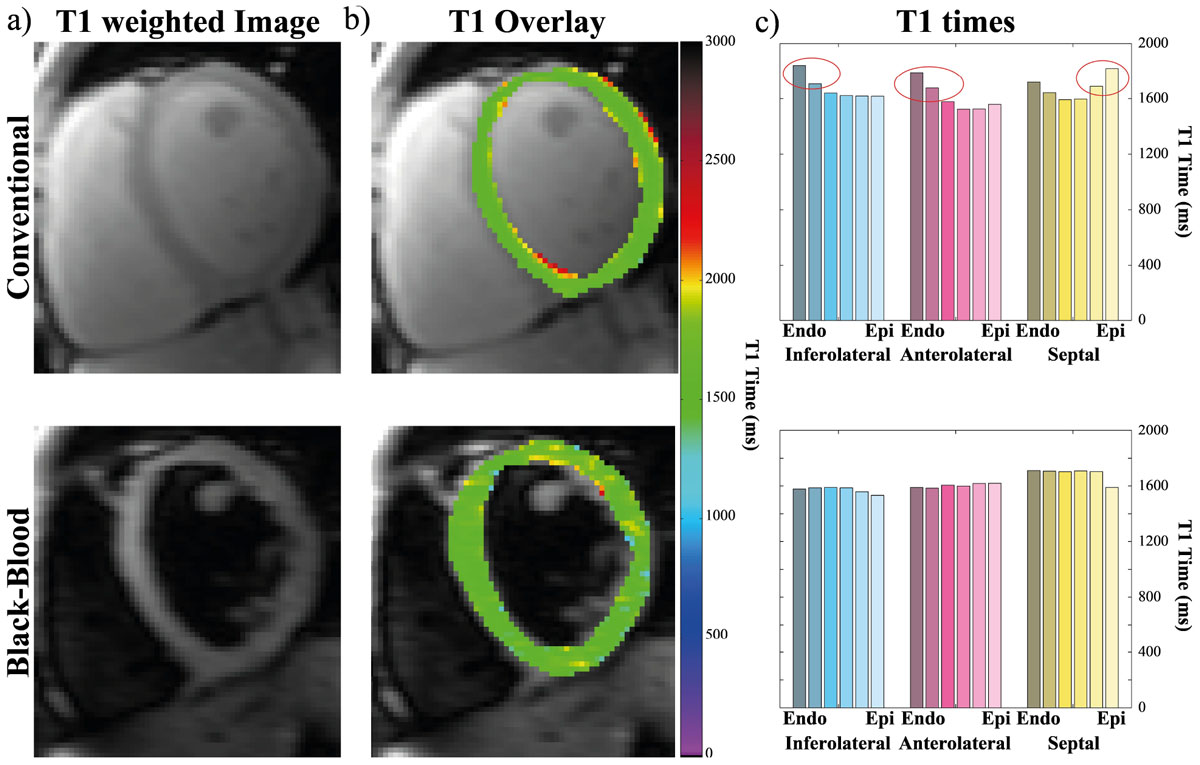
**a) T**_**1**_**-weighted images of a healthy volunteer with and without MSDE magnetization preparation**. Visually strong and homogenous suppression of the blood-signal can be observed. b) Corresponding T_1_ quantification in the myocardium using conventional and black-blood T_1_ mapping. Visually homogenous T_1_ times are observed around the myocardium using both methods. c) T_1_ times through the myocardial thickness analyzed separately for the anterolateral, inferolateral and septal part of the myocardium. Conventional T_1_ mapping shows strongly elevated T_1_ times at the endo- and/or the epicardial border. No such elevation is observed with black-blood T_1_ mapping.

Visually strong blood suppression was achieved using the adiabatic MSDE preparation (Fig. 2a). Quantitative analysis reveals increased T_1_ times towards the myocardial borders in conventional T_1_ mapping (Figure 2c), while consistent T_1_ times through the entire myocardial thickness were measured using black-blood SAPPHIRE. No significant difference was found in the average T_1_ time of the two methods (Conv.: 1574 ± 52 ms vs BB: 1593 ± 47 ms). A 25%-28% gain in apparent in-slice area of the myocardium and average wall-thickness in the T_1_ maps was achieved using blood-suppression (BB: 1596 ± 266 mm^2^, 7.37 ± 1.16 mm vs. Conv.: 1278 ± 213 mm^2^, 5.72 ± 0.87 mm, p < 0.05).

## Conclusions

An adiabatic MSDE preparation enables robust myocardial T_1_ Mapping at 3T. The apparent myocardial in-slice area and average wall-thickness is significantly increased using a black-blood preparation. Furthermore, elevated T_1_ times at the myocardial borders were eliminated. This reduces sensitivity to ROI placement and potentially benefits the reproducibility of myocardial T_1_ mapping, especially in the presence of pathologies with reduced myocardial wall-thickness.

